# The virological durability of first-line ART among HIV-positive adult patients in resource limited settings without virological monitoring: a retrospective analysis of DART trial data

**DOI:** 10.1186/s12879-017-2266-3

**Published:** 2017-02-21

**Authors:** David I. Dolling, Ruth L. Goodall, Michael Chirara, James Hakim, Peter Nkurunziza, Paula Munderi, David Eram, Dinah Tumukunde, Moira J. Spyer, Charles F. Gilks, Pontiano Kaleebu, David T. Dunn, Deenan Pillay, on behalf of the DART Virology Group, P. Kaleebu, D. Pillay, P. Awio, M. Chirara, D. Dunn, D.M. Gibb, C. Gilks, R. Goodall, A. Kapaata, M. Katuramur, F. Lyagoba, B. Magambo, K. Mataruka, L. Mugarura, T. Musunga, M. Nabankkema, J. Nkalubo, P. Nkurunziza, C. Parry, V. Robertson, M. Spyer, D. Mulima, D.E. Williams, I. Nankya, S. Nassimbwa, E. Ndashimye, E. Nabulime, M. Phiri, K. Mutasa, S. Mukasa

**Affiliations:** 1 MRC Clinical Trials Unit at UCL, London, UK; 2 University of Zimbabwe, Harara, Zimbabwe; 3 Joint Clinical Research Centre, Kampala, Uganda; 4 MRC/UVRI Uganda Research Unit on AIDS, Entebbe, Uganda; 5 School of Population Health, University of Queensland, Brisbane, Australia; 6 Africa Centre for Health and Population Studies, University of KwaZulu Natal, Durban, South Africa

**Keywords:** Treatment outcomes, HIV-infected adults, Virological failure, Resource-limited, Low-income

## Abstract

**Background:**

Few low-income countries have virological monitoring widely available. We estimated the virological durability of first-line antiretroviral therapy (ART) after five years of follow-up among adult Ugandan and Zimbabwean patients in the DART study, in which virological assays were conducted retrospectively.

**Methods:**

DART compared clinically driven monitoring with/without routine CD4 measurement. Annual plasma viral load was measured on 1,762 patients. Analytical weights were calculated based on the inverse probability of sampling. Time to virological failure, defined as the first viral load measurement ≥200 copies/mL after 48 weeks of ART, was analysed using Kaplan-Meier plots and Cox regression models.

**Results:**

Overall, 65% of DART trial patients were female. Patients initiated first-line ART at a median (interquartile range; IQR) age of 37 (32–42) and with a median CD4 cell count of 86 (32–140). After 240 weeks of ART, patients initiating dual-class nucleoside reverse-transcriptase inhibitor (NRTI) -non-nucleoside reverse-transcriptase (NNRTI) regimens containing nevirapine + zidovudine + lamivudine had a lower incidence of virological failure than patients on triple-NRTI regimens containing tenofovir + zidovudine + lamivudine (21% vs 40%; hazard ratio (HR) =0.48, 95% CI:0.38–0.62; *p* < 0.0001). In multivariate analyses, female patients (HR = 0.79, 95% CI: 0.65–0.95; p = 0.02), older patients (HR = 0.73 per 10 years, 95% CI: 0.64–0.84; *p* < 0.0001) and patients with a higher pre-ART CD4 cell count (HR = 0.64 per 100 cells/mm^3^, 95% CI: 0.54–0.75; *p* < 0.0001) had a lower incidence of virological failure after adjusting for adherence to ART. No difference in failure rate between the two randomised monitoring strategies was observed (*p*= 0.25).

**Conclusions:**

The long-term durability of virological suppression on dual-class NRTI-NNRTI first-line ART without virological monitoring is remarkable and is enabled by high-quality clinical management and a consistent drug supply. To achieve higher rates of virological suppression viral-load-informed differentiated care may be required.

**Trial Registration:**

Prospectively registered on 18/10/2000 as ISRCTN13968779.

**Electronic supplementary material:**

The online version of this article (doi:10.1186/s12879-017-2266-3) contains supplementary material, which is available to authorized users.

## Background

The treatment of human immunodeficiency virus (HIV) in low-income countries has predominantly followed a “public health approach” as access to antiretroviral therapy (ART) has been scaled up [1, 2]. This approach recommends standardised treatment regimens and a simplified approach for monitoring patients on ART. The World Health Organisation (WHO) guidelines for the treatment of HIV are regularly updated and in 2010 [3] recommended that patients receive regular clinical and immunological monitoring whilst on treatment. Additionally, if resources permit, they advised that viral load should be used in a targeted approach to confirm treatment failure detected either immunologically or clinically. The 2013 guidelines [4] were updated to strongly recommend HIV viral load monitoring six months after initiating ART and then every twelve months, although noted that this strong recommendation was based on low-quality evidence. Despite a paucity of evidence, there remains a widespread anxiety that without virological monitoring patients may remain on a treatment regimen that they are virologically failing for a sustained period of time. This could lead to worse long-term clinical outcomes and potentially the accumulation of drug-resistance mutations that compromise second-line ART (although it is noteworthy that substantial cross-resistance did not impair response to second-line in the EARNEST trial [5]). Furthermore, there are concerns that immunological criteria for switching treatment have low specificity and can lead to unnecessary treatment switches to more expensive second-line regimens, which in low-income settings may be the last available treatment option.

Individual countries are deciding, within financially-restricted healthcare systems, whether they should invest in upgrading laboratory infrastructure to facilitate virological monitoring. Currently, despite 39/52 low and middle-income countries recommending viral load testing, in only 8/52 has testing become widely available [6]. Cost-effectiveness studies have evaluated the potential trade-offs between expanding access to ART to more patients, viral load versus CD4 monitoring and alternative monitoring frequencies for patients and through viral-load-informed differentiated care [7]. Keebler et al. [8] concluded that “viral load monitoring should only be considered after high antiretroviral therapy coverage has been achieved”, for example by raising the CD4 threshold at which ART is initiated. More recently, a working group on modelling of ART monitoring strategies in Sub-Saharan Africa [7] found that through the use of dried blood sample testing and tailored care, such as patients with suppressed viral load visiting clinics less frequently, a cost-effective strategy was possible. A critical parameter for cost-effectiveness models is the rate of virological failure. To date, this has been entirely estimated from cohorts that received regular viral load monitoring, which may not reflect the experience of the majority of patients who have been treated in settings without laboratory monitoring.

In this analysis, we report longitudinal findings from the DART trial on the virological durability of first-line ART over 240 weeks of follow-up among adult Ugandan and Zimbabwean patients without real-time viral load monitoring.

## Methods

### Study overview

DART was a randomised open-label non-inferiority trial conducted in ART-naïve, symptomatic HIV-infected adults with a CD4 cell count ≤200 cells/mm^3^. DART enrolled from three clinical centres in Uganda and one in Zimbabwe. Recruitment was between January 2003 and October 2004 and patients were followed until the end of 2008 [9]. Patients were randomised to clinically-driven monitoring only (CDM) or clinical monitoring plus routine laboratory monitoring (LCM) in the form of twelve-weekly CD4 and haematological/biochemical toxicity tests. CD4 cell count results were not returned to patients in the CDM arm. At each visit a plasma sample was stored; real-time viral load monitoring was not conducted during DART. Patients were switched to a second-line regimen containing a ritonavir-boosted protease inhibitor (PI) if a new or recurrent WHO stage 4 event occurred. Alternatively, patients could switch at the clinician’s discretion if one or more WHO stage 3 events occurred (such as candidiasis or weight loss). Additionally, in the LCM arm, patients could switch treatment if there was a confirmed CD4 cell count <100 cells/mm^3^ (<50 cells/mm^3^ prior to July 2006). Switching treatment before 48 weeks was strongly discouraged for patients on both monitoring strategies. The DART trial was approved by research ethics committees in Uganda, Zimbabwe, and the UK. Patient consent included the storage of plasma samples and later testing.

Three different first-line ART regimens were used in the DART study. All patients received co-formulated zidovudine (ZDV) and lamivudine (3TC). In a Ugandan substudy [10], 600 patients were randomised to receive either abacavir (ABC, *n* = 300) or nevirapine (NVP, *n* = 300). An additional 247 patients in Zimbabwe received open-label NVP. All other patients (*n* = 2,469) received a triple-nucleoside reverse-transcriptase inhibitor (NRTI) regimen, which included the third drug tenofovir (TDF).

### Sample storage

Whole bloods were collected in EDTA vacutainers at routine visits during DART follow up and plasma isolated by centrifugation within 2 to 6 h following local laboratory procedures. Plasma was distributed into 2 ml cryovials as 1 ml to 1.5 ml aliquots and stored frozen at minus 80 °C until retrieval for HIV viral load assays.

### Sample selection

Viral load testing was conducted on approximately 3,500 of the 60,000 plasma samples held in the DART repository. Patients were excluded completely if (a) they either died or switched treatment prior to week 48, since early deaths were unlikely to be due to treatment failure [11] and early switches were rare and discouraged, or (b) received a structured treatment interruption [12], because these are no longer recommended in treatment guidelines. All other patients who received either NVP (*n* = 404) or ABC (*n* = 254) were selected, as well as a sample of patients who received TDF (*n* = 1,104). This sample selectively included patients who received a viral load test under a national Ugandan viral load testing program shortly after trial closure [13]. Further details on the sampling process are included in Additional file 1.

Plasma samples for viral load testing from this sample of patients were selected using a “walkback” procedure, beginning with the last sample on first-line ART. If viral load was <200 copies/mL no further samples were tested; if not, a sample from approximately 48 weeks earlier was tested. This process was repeated at approximate 48-week intervals until a result <200 copies/mL was obtained or until the week 48 sample was tested. In addition, samples from baseline (week 0) were tested in 74% of patients; the 24% baseline samples not tested were from those on TDF known to be suppressed at the end of the study via the national Ugandan testing program.

### Statistical methods

To correct for non-random sampling, analytical probability weights based on the inverse probability of a patient being sampled, were used in all analyses to reflect the DART population as a whole [14]. Time to persistent virological failure, defined as the first viral load measurement ≥200 copies/mL after 48 weeks of ART, was analysed using adjusted Kaplan-Meier estimators and Cox regression models (stratified by study centre) incorporating the analytical weights [15]. Non-proportionality was investigated using Schoenfeld residuals and continuous variables were included in the Cox model using a multivariable fractional polynomial model [16]. In the primary analysis, patients were censored at the time of switch to second-line ART or death if they had not virologically failed by this time. In a sensitivity analysis we considered all deaths and ART switches after 48 weeks of ART as virological failures regardless of their viral load at the time of the event. A second sensitivity analysis considered all deaths and ART switches as virological failures. A final sensitivity analyses investigated the effect of alternative definitions of virological failure. Adherence was included as a time-dependent covariate summarizing the estimated adherence in each 48 week period, as measured by the proportion of visits where pill counts indicated greater than 95% drug possession ratio (defined as the days’ supply of drugs delivered minus the days’ supply of drugs returned divided by the number of days between clinic visits) [17].

To avoid a loss in efficiency, missing values for baseline viral loads on the log_10_ scale were multiply imputed 30 times using a linear regression model which included all potential prognostic factors (terms of Cox model) and outcome variables (Nelson-Aalen estimator for time to virological failure, censoring indicator) [18]. This assumes that missing values do not depend on unobserved variables conditional on the observed data, such as the outcome variable. Analyses were performed on each imputed dataset and the imputation-specific coefficients combined using Rubin’s rules. All analyses were conducted using Stata 13.1 [19].

## Results

### Baseline characteristics

Baseline characteristics for patients included in the overall DART trial and viral load substudy are shown in Table 1. Overall, 65% of DART trial patients were female. Patients initiated first-line ART at a median (interquartile range (IQR)) age of 37 (32–42) years and with a median CD4 cell count of 86 (32–140) cells/mm^3^. Overall 17% of patients received a dual-class NRTI/non-NRTI (NNRTI) regimen and 83% received a triple-NRTI regimen. Characteristics were broadly similar in the viral load substudy apart from first-line ART (patients prescribed NVP or ABC over-represented in the substudy by design) and study centre (Kampala over-represented and Harare under-represented by design). However, the use of analytical weights (see Statistical methods) corrects for these design imbalances.

**Table 1:** Patient characteristics, overall and patients sampled for viral load substudy

Factor	Overall	Viral load substudy
Monitoring randomisation		
LCM	1,502 (50%)	882 (50%)
CDM	1,505 (50%)	880 (50%)
Gender		
Male	1,051 (35%)	587 (33%)
Female	1,956 (65%)	1,175 (67%)
Age at ART initiation (years) Median (IQR)	37 (32 – 42)	37 (32 – 43)
CD4 at ART initiation (cells/mm^3^)		
Median (IQR)	86 (32 – 140)	83 (31 – 137)
0-49	996 (33%)	585 (33%)
50-99	725 (24%)	440 (25%)
100-149	687 (23%)	400 (23%)
150-200	599 (20%)	337 (19%)
Viral load at ART initiation (copies/mL)		
Missing	-	452 (26%)
<30,000	-	132 (10%)
30,000 – 100,000	-	181 (10%)
100,000 – 300,000	-	373 (21%)
300,000 – 700,000	-	340 (19%)
>700,000	-	284 (16%)
First-line ART^a^		
TDF	2,196 (73%)	1,104 (63%)
NVP	520 (17%)	404 (23%)
ABC	291 (10%)	254 (14%)
TB in 12 months prior to enrolment	737 (25%)	421 (24%)
Centre		
Entebbe, Uganda	914 (30%)	543 (31%)
Kampala, Uganda	1,159 (39%)	809 (46%)
Harare, Zimbabwe	934 (31%)	410 (23%)
Adherence at week 48		
0 – 50%	106 (4%)	68 (4%)
50 – 67%	175 (6%)	108 (6%)
67 – 75%	251 (8%)	152 (9%)
75 – 83%	456 (15%)	264 (15%)
83 – 92%	876 (29%)	523 (30%)
>92%	1,134 (38%)	645 (37%)
Missing	9 (0%)	2 (0%)

^a^In conjunction with co-formulated AZT/3TC

### Durability of virological suppression

**Fig. 1:**
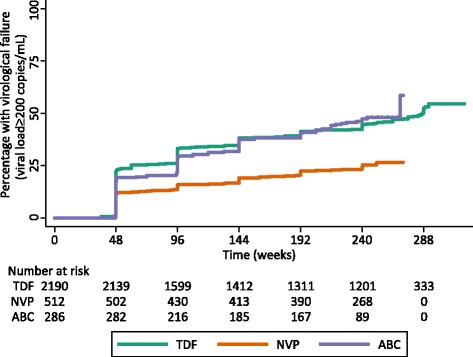
Cumulative percentage with virological failure (viral load > 200 copies/mL) by first-line ART regimen. Figure shows cumulative percentage with virological failure estimated using the Kaplan-Meier method incorporating analytical weights. Number at risk = number of patients alive and on continuous first-line ART without virological failure

Overall, one or more viral load measurements were available for 1,741/1,762 (99%) patients. The first sample tested (last time point on first-line ART) was after a median (IQR) 252 (224–280) weeks on first-line ART. In total, 606 patients (35%) were observed to fail virologically during follow-up. Figure 1 displays the cumulative percentage with virological failure over time, by first-line ART received. At 48 weeks, 19% (95% confidence interval (CI): 17–20%) of patients had virological failure - approximately half of all failures which occurred by 240 weeks. Most treatment switches occurred with virological failure (83%) and second-line switches with treatment failure were more likely in the LCM arm (128/145; 88% compared to 94/122; 77%; *p* = 0.02). Overall, an estimated 28% (95% CI: 26–29%) of patients had experienced virological failure by 96 weeks, 32% (95% CI: 31–34%) by 144 weeks, 35% (95% CI: 33-37%) by 192 weeks and 37% (95% CI: 35–39%) by 240 weeks. The rate of virological failure was similar for patients receiving triple-NRTI regimens containing either TDF or ABC (40% (95% CI: 38–42%) and 45% (95% CI: 38–50%) respectively at 240 weeks; *p* = 0.37), but was substantially lower for patients who received a dual-class regimen containing NVP (21% (95% CI: 18–25%) at 240 weeks; p < 0.0001).

**Fig. 2:**
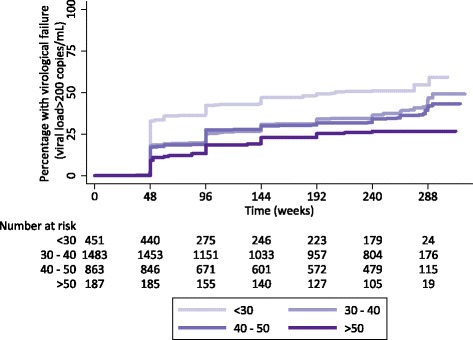
Cumulative percentage with virological failure (viral load > 200 copies/mL) by age at randomisation. Figure shows cumulative percentage with virological failure estimated using the Kaplan-Meier method incorporating analytical weights. Number at risk = number of patients alive and on continuous first-line ART without virological failure

There was no evidence that monitoring strategy affected long-term virological failure in the multivariable Cox model (Table 2) (*p* = 0.25). However, both gender and age were strong predictors of virological failure. Female patients had a 21% lower incidence of virological failure (*p* = 0.01) and each additional 10 year increase in a patient’s age reduced incidence of virological failure by 27% (Fig. 2; *p* < 0.0001). Each additional 100 cell increase in patient’s baseline (pre-ART) CD4 cell count reduced incidence of virological failure by 36% (*p* < 0.0001). There was no evidence that baseline viral load affected the incidence of virological failure (*p* = 0.89). The multivariable fractional polynomial model revealed no evidence of non-linearity and there was no evidence of non-proportionality for either monitoring strategy or initial ART. Compared with the TDF reference group, patients who received a NVP-containing regimen had a 52% (*p* < 0.0001) lower incidence of virological failure and patients prescribed ABC a 27% (*p* = 0.03) higher incidence of virological failure. Adherence, as measured by the proportion of visits in the past 48 weeks where the drug possession ratio was >95%, showed clear association with virological failure, with 11% lower incidence of virological failure for every 10% increase in the proportion of adherent visits (*p* < 0.0001).

**Table 2:** Cox regression analysis of predictors of virological failure

Factor	Univariable HR	*p*-value	Multivariable HR (95% CI)	*p*-value
Monitoring randomisation				
LCM	1.00	-	1.00	-
CDM	1.07	0.45	1.11 (0.93 - 1.35)	0.25
Gender				
Male	1.00	-	1.00	-
Female	0.80	0.02	0.79 (0.65 - 0.95)	0.01
Initial ART				
TDF	1.00	<0.0001	1.00	<0.0001
NVP	0.49	-	0.48 (0.38 - 0.62)	-
ABC	1.18	-	1.27 (1.02 - 1.59)	-
TB in 12 months prior to enrolment	1.13	0.26	1.07 (0.86 - 1.33)	0.52
Age (per 10 years older)	0.73	<0.0001	0.73 (0.64 - 0.84)	<0.0001
Baseline CD4 (per 100 cells/mm^3^ higher)	0.60	<0.0001	0.64 (0.54 - 0.75)	<0.0001
Baseline viral load (log_10_ copies/mL)	1.02	0.84	1.01 (0.84 - 1.22)	0.89
Adherence in previous 48 weeks^a^ (per 10% higher)	0.89	<0.0001	0.89 (0.84 - 0.94)	<0.0001

^a^time-updated

Previous research suggests that ABC-based regimens may be more prone to failure for baseline viral loads ≥ 100,000 copies/mL [20]. A test for interaction indicated that there was an association between initial ART and a baseline viral load ≥ 100,000 copies/mL in this data (*p* = 0.02). Patients on ABC with a baseline viral load < 100,000 copies/mL had a 48% (95% CI: 18–68%) lower incidence of virological failure compared to those with a baseline viral load ≥ 100,000 copies/mL. However, this effect was much less marked for patients who received TDF (20% lower incidence, 95% CI −8–41%) or NVP (13% lower incidence, 95% CI −41–46%).

### Sensitivity analyses

The first sensitivity analysis, which considered all deaths and ART switches (*n* = 86) after 48 weeks as virological failures, slightly increased the cumulative incidence of virological failure (41% by 240 weeks) but had a negligible effect on the multivariable hazard ratios (HR) (Table 3). Secondly, assuming that deaths and ART switches prior to week 48 (*n* = 172) were also virological failures increased the overall cumulative incidence of virological failure to 41% by 240 weeks and for patients on dual-class regimens containing nevirapine to 24%. Finally, alternative thresholds for virological failure were investigated. Using a threshold of >1,000 copies/mL did not materially change our findings but increasing the threshold to >10,000 copies/mL led to a similar incidence of virological failure for patients on ABC compared to TDF. This suggests that the differences in failure rates between patients on ABC and TDF are driven by a higher proportion of patients on ABC failing with a viral load between 200 and 10,000 copies/mL.

**Table 3:** Sensitivity analyses of virological failure definition

	Default		Sensitivity 1		Sensitivity 3			
	VF ≥ 200 cps/mL		VF ≥ 200 cps/mL		VF ≥ 1,000 cps/mL		VF ≥ 10,000 cps/mL	
	Multivariable HR (95% CI)	*p*-value	Multivariable HR (95% CI)	*p*-value	Multivariable HR (95% CI)	*p*-value	Multivariable HR (95% CI)	*p*-value
Monitoring randomisation								
LCM	1.00	-	1.00	-	1.00	-	1.00	-
CDM	1.11 (0.93 – 1.34)	0.25	1.16 (0.98 – 1.38)	0.09	1.14 (0.94 – 1.37)	0.19	1.18 (0.96 – 1.45)	0.11
Gender								
Male	1.00	-	1.00	-	1.00	-	1.00	-
Female	0.79 (0.65 – 0.95)	0.01	0.84 (0.70 – 1.01)	0.06	0.78 (0.64 – 0.95)	0.01	0.77 (0.62 – 0.95)	0.02
Initial ART								
TDF	1.00	<0.0001	1.00	<0.0001	1.00	<0.0001	1.00	<0.0001
NVP	0.48 (0.38 – 0.62)	-	0.49 (0.39 – 0.62)	-	0.49 (0.38 – 0.63)	-	0.50 (0.38 – 0.65)	-
ABC	1.27 (1.02 – 1.59)	-	1.23 (0.99 – 1.52)	-	1.23 (0.97 – 1.55)	-	1.00 (0.76 – 1.31)	-
TB in 12 months prior to enrolment	1.07 (0.86 – 1.33)	0.52	1.07 (0.88 – 1.32)	0.47	1.08 (0.86 – 1.36)	0.49	1.15 (0.91 – 1.46)	0.24
Age (per 10 years older)	0.73 (0.64 – 0.84)	<0.0001	0.77 (0.68 – 0.88)	<0.0001	0.70 (0.61 – 0.81)	<0.0001	0.69 (0.60 – 0.80)	<0.0001
Baseline CD4 (per 100 cells/mm^3^ higher)	0.64 (0.54 – 0.75)	<0.0001	0.67 (0.57 – 0.78)	<0.0001	0.60 (0.50 – 0.72)	<0.0001	0.52 (0.43 – 0.64)	<0.0001
Baseline viral load (log_10_ copies/mL)	1.01 (0.84 – 1.22)	0.89	0.99 (0.83 – 1.18)	0.91	1.02 (0.84 – 1.24)	0.82	1.02 (0.82 – 1.25)	0.88
Adherence in previous 48 weeks^a^ (per 10% higher)	0.89 (0.84 – 0.94)	<0.0001	0.90 (0.85 – 0.94)	<0.0001	0.89 (0.84 – 0.94)	<0.0001	0.88 (0.82 – 0.94)	<0.0001

^a^time-updated

## Discussion

This analysis found that at 240 weeks an estimated 79% of patients on dual-class regimens in the DART study were virologically suppressed. This was particularly noteworthy given that the overall median CD4 cell count when ART was initiated was 86 cells/mm^3^ and a third had <50 cells/mm^3^. Persistent undetected virological failure was relatively low (20%); high virological failure rates are not an inevitable consequence of using only clinical or CD4 cell count monitoring. Within financially-restricted healthcare systems the lack of availability of virological monitoring should not limit countries when expanding access to ART. However, to achieve higher rates of virological suppression, viral-load-informed differentiated care may be required [7].

The rate of virological failure in patients who started ART with a triple-NRTI regimen was approximately twice that of dual-class regimens. Baseline CD4 cell count remained a strong predictor of virological failure after 48 weeks on first-line ART. Despite adjusting for differences in adherence, both female patients and older patients had a lower incidence of virological failure.

### Comparisons with other studies

A previous cross-sectional analysis of data from Ugandan centres at the end of the DART study found 80% of patients who remained on first-line ART had suppressed viral load (<400 copies/mL) [13]. Virological suppression was also shown to differ by trial arm (*p* = 0.003), 76% were suppressed in the CDM arm compared to 83% in the LCM arm. Our research extends this study by including longitudinal data, data from Zimbabwe and patients who switched treatment or died during the study. Importantly, more patients in LCM switched to second-line and were excluded from this previous analysis: these patients were also more likely to virologically fail [21]. After including the greater proportion of patients with virological failure in the LCM arm who switched to second-line ART we demonstrated no overall difference by randomised monitoring strategy in long-term virological failure.

Even though most patients were receiving triple NRTIs first-line, we found more durable virological suppression than a 2015 meta-analysis [22], in which 62% (*n* = 504) of adult patients on NNRTI-based regimens in four studies from low and middle income countries remained virologically suppressed in an intention-to-treat analysis after 48 months of ART. Of those on treatment, 83% (*n* = 909) of patients from six studies were virologically suppressed after 60 months of ART. Unlike our analysis, both of these estimates did not consider switches to second-line ART as virological failure. Cost-effectiveness analyses conducted by Phillips et al. [23] assume that 25% of patients starting ART for the first time with a boosted PI or NNRTI regimen virologically fail by 5 years, slightly higher than our findings for patients on dual-class regimens containing NVP.

We show that patients on ABC have a higher incidence of virological failure than patients on TDF, in line with previous research [20, 24] and a meta-regression analysis [25] comparing these drugs in dual-class regimens containing NNRTI or PIs [20, 24, 26]. Sax et al. [20] found that there was no difference in time to virological failure in patients on dual-class regimens with baseline viral load < 100,000 copies/mL, but that there was a shorter time to failure in patients on ABC with baseline viral load > 100,000 copies/mL. Our analysis shows that this remains true in triple-NRTI regimens and supports treatment guidelines which recommend that ABC is only used when baseline viral load is <100,000 copies/mL.

Some studies [25, 27] have demonstrated that older patients have a better virological response to ART, although many other studies have found no effect of age [25]. An improved virological response is often speculated to be a result of greater adherence in older patients [25]. The age effect in DART persisted after controlling for a measure of adherence based on pill counts and returns. However, adherence may be under reported by age so pharmacologic effects such as decreased metabolism with older age cannot be excluded.

Women were shown to have significantly lower incidence of virological failure than men after adjusting for differences in adherence, which has not been widely shown before. A recent systematic review on sex differences in HIV outcomes [28] reported a non-significant, marginally decreased risk of virological failure for female patients (pooled risk ratio = 0.93; 95% CI = 0.85 – 1.01). However, a meta-analysis of randomised trials found no difference in virological outcome at week 48 [29]. Inaccurate reported adherence may account for some of this difference although pharmacologic effects related to gender cannot be excluded.

There was no evidence that baseline viral load was associated with the incidence of virological failure. In contrast, a meta-analysis [30] has demonstrated that patients with baseline viral load >100,000 copies/mL are less likely to be virologically suppressed after 48 weeks of treatment. The lack of association in DART could be due to the high viral load at baseline (24% of patients in DART had a baseline viral load ≤ 100,000 copies/mL compared to 55% in Stephan et al. [30]) and may indicate that the effect plateaus above 100,000 copies/mL. Additionally, it is also possible that with increased follow-up the effect of baseline characteristics is diminished. However, Stephan et al. [30] note that even among the 21 trials they examined there are inconsistent results for baseline viral load.

### Study limitations

The majority of patients in DART used triple-NRTI regimens which are no longer used in low-income countries so findings for these patients are of less interest and included for completion. Nonetheless, this analysis contains virological outcome data for 404 patients who used a dual-class regimen containing nevirapine with 240 weeks of follow-up data. The recent systematic review [21] only contained intention-to-treat data for 504 patients after 24 months of ART so this data contributes substantially to the existing evidence. Since the DART trial was conducted in 2010 there has been increased access to ART in low-income settings and growing ART-naïve HIV drug resistance. This could increase the value of viral load monitoring but is not evaluable in the DART trial population.

Due to funding, only a proportion of patients in DART could have virological testing conducted. This should not bias the results since the complete analysis population of interest on nevirapine and abacavir were sampled and a random sample was taken of patients on tenofovir. Patients were appropriately weighted in all analyses to account for the sampling method. It is likely that including patients who died prior to week 48 would increase the proportion detected with virological failure.

The walkback approach to viral load sampling meant that intermittent or early virological failure in patients who subsequently re-suppressed is not detected. The clinical consequences of intermittent viremia in low-income countries are complex but with limited availability of second-line regimens in low-income countries the priority is to achieve long-term virological suppression on first-line regimens. Including patients with intermittent or virological failure before 48 weeks of ART would increase the proportion detected with virological failure.

DART was a randomised clinical trial with a high quality of clinical care, so it could be argued that the sustained virological suppression observed is not generalizable to wider clinical settings in resource-limited countries. Nonetheless, this analysis demonstrates that long-term virological suppression on dual-class regimens containing nevirapine is feasible if patients receive regular clinic visits and have consistent drug supply, despite the absence of laboratory monitoring, sub-optimal treatment regimens and low baseline CD4 cell count.

Analyses of drug resistance assays on the samples in this study are currently ongoing. Therefore at this stage we are not able to conclude whether virological failure is the result of non-adherence to ART or due to HIV-1 drug resistance mutations developing. Similarly, the clinical consequences of virological failure are also being investigated in ongoing analyses.

## Conclusions

Our analysis longitudinally examines virological failure in a setting without virological monitoring over five years of follow-up. The rate of virological failure is lower than a recent systematic review, particularly given that most patients received less potent triple-NRTI regimens. Our results suggest that within financially-restricted healthcare systems, virological control is achievable in the absence of real time viral load testing, and that a consistent drug supply, enabling high levels of adherence, is likely more crucial for sustained virological suppression than upgrading clinical and laboratory infrastructure. To achieve higher rates of long-term virological suppression, viral-load informed differentiated care may be required.

## Additional files

Additional file 1: Selection Process. (DOCX 334 kb)

## Abbreviations

3TC: Lamivudine
ABC: Abacavir
ART: Antiretroviral therapy
CDM: Clinically-driven monitoring only
CI: Confidence interval
EDTA: Ethylenediaminetetraacetic acid
HIV: Human immunodeficiency virus
HR: Hazard ratio
IQR: Interquartile range
LCM: Clinical monitoring plus routine laboratory monitoring
NNRTI: Non-nucleoside reverse-transcriptase inhibitor
NRTI: Nucleoside reverse-transcriptase inhibitor
NVP: Nevirapine
PI: Protease inhibitor
TDF: Tenofovir
WHO: World Health Organisation
ZDV: Zidovudine
