# Supplementary material for: The virological durability of first-line ART among HIV-positive adult patients in resource limited settings without virological monitoring: a retrospective analysis of DART trial data
Source: BMC Infect Dis. 2017 Feb 21;17:160. doi: 10.1186/s12879-017-2266-3 (PMC5319022; doi:10.1186/s12879-017-2266-3)
Supplement: Additional file 1: — Selection Process. (DOCX 334 kb) [file 12879_2017_2266_MOESM1_ESM.docx]

Additional File 1

Selection Process

Firstly, patients were not tested if they either died (n=171) or switched treatment (n=1) prior to week 48; since early deaths were unlikely to be due to treatment failure[^8^](#_ENREF_8) and early switches were rare and strongly discouraged in the protocol. Secondly, patients who received a structured treatment interruption were excluded because these are no longer recommended in treatment guidelines. Structured treatment interruptions were part of a previously published separate substudy (DART Trial Team, AIDS 2008: 22:237-247) where after 48 or 72 weeks patients with CD4 cell counts ≥ 300 cells/mL were randomised to continuous treatment or 12 weekly cycles on/off treatment.

All other patients who received either NVP (n=404) or ABC (n=254) were selected to offer maximum power in comparison to patients on TDF. For the remaining TDF patients, a more complicated sampling scheme was used. Firstly, all patients who died after 48 weeks were sampled. Secondly, a random sample of 90 patients who switched treatment during the study (30 from each centre) was chosen. Thirdly, all patients from the earlier virology substudy (part I/II) were sampled because these patients had existing viral loads and resistance sequences for baseline and week 48 which could be utilised. Finally, all Ugandan patients with a first-line viral load results available from the national Ugandan TREAT program were sampled. This group consisted of 442 patients with a viral load<200 copies/mL at the end of the study and 140 with a detectable viral load. An additional random sample of 70 Zimbabwean patients, who were on first-line treatment at the end of study follow-up and who had been recruited before 1^st^ January 2004, was also chosen to achieve approximately 70 patients with a detectable viral load from each treatment centre. In total 1,762 patients were sampled.


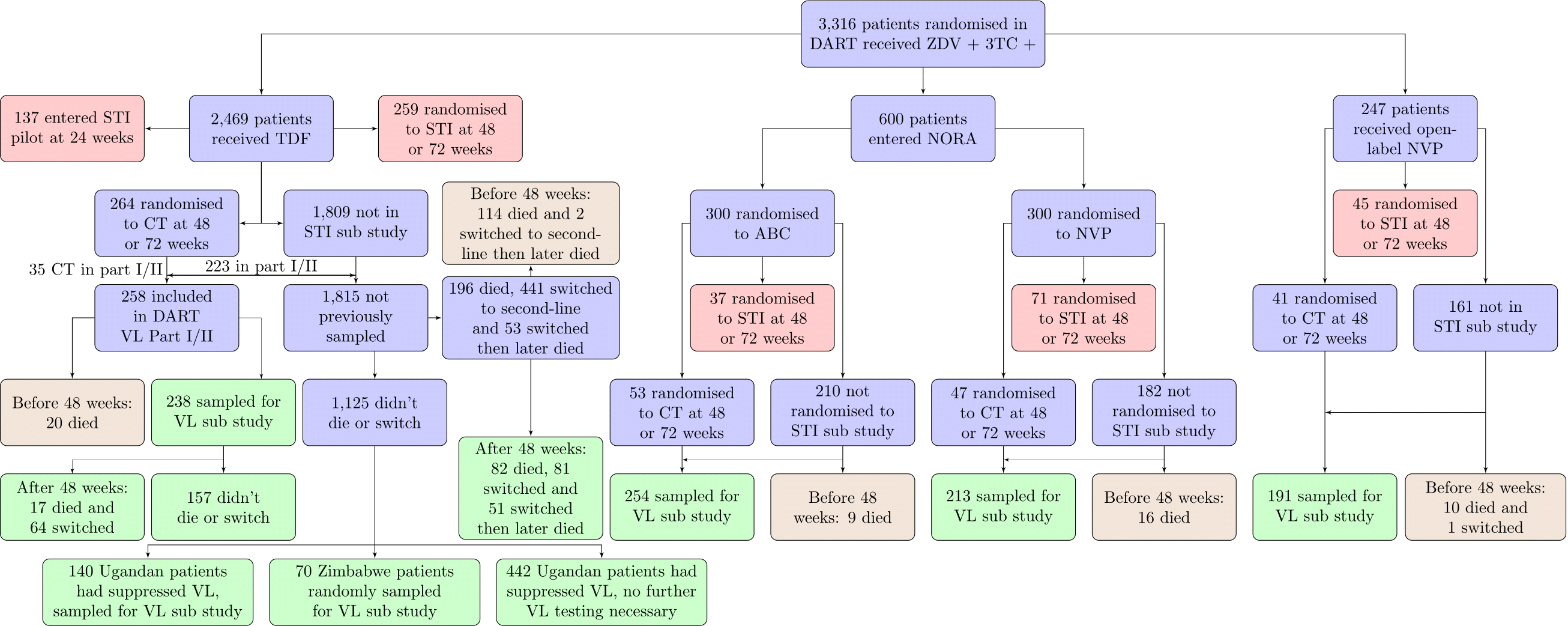


Additional Figure 1: DART virology selection process
